# Supplementary material for: Mechanism of Takifugu bimaculatus Skin Peptides in Alleviating Hyperglycemia in Rats with Type 2 Diabetic Mellitus Based on Microbiome and Metabolome Analyses
Source: Mar Drugs. 2024 Aug 22;22(8):377. doi: 10.3390/md22080377 (PMC11355842; doi:10.3390/md22080377)
Supplement: Supplementary file 1 [file marinedrugs-22-00377-s001.zip › marinedrugs-3127749-supplementary.pdf]

Supplementary information

Mechanism of *Takifugu bimaculatus* Skin Peptides in Alleviating Hyperglycemia in Rats with Type 2 Diabetic Mellitus Based on Microbiome and Metabolome Analyses

Min Xu [1,2], Bei Chen [2], Kun Qiao [2], Shuji Liu [2], Yongchang Su [2], Shuilin Cai [2], Zhiyu Liu [2,*] Lijun Li [1,*] and Qingbiao Li [1,*]

1 College of Ocean Food and Bioengineering, Jimei University, Xiamen. 361021, China; xumin1315@foxmail.com (M.X.)

2 Key Laboratory of Cultivation and High-Value Utilization of Marine Organisms in Fujian Province, Fisheries Research Institute of Fujian, Xiamen 361013, China; chenbeifjfri@foxmail.com (B.C.); qiaokun@qiaokun@xmu.edu.cn (K.Q.); cute506636@163.com (S.L.); suyongchang@stu.hqu.edu.cn (Y.S.); caishuilin@hqu.edu.cn (S.C.)

* Correspondence: negroliu@163.com (Z. L); ljli@jmu.edu.cn (L. L); qbli@jmu.edu.cn (Q. L)

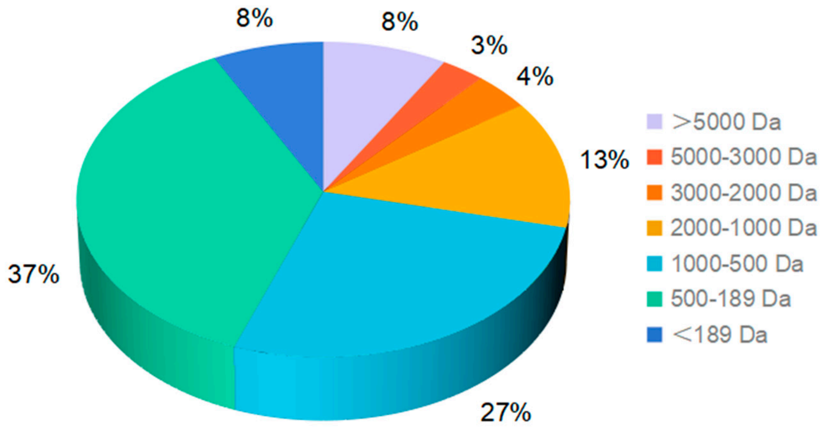

Figure S1. Molecular weight distribution of TBSH.

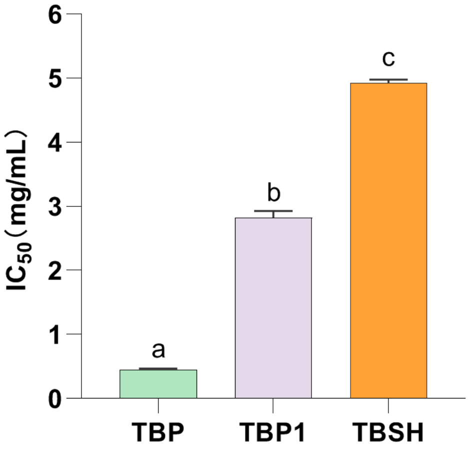

Figure S2. DPP-IV inhibition IC_{50} of TBP (Mw <1 kD), TBP1 (Mw ≥1 kD), and hydrolysate on Takifugu bimaculatus skin (TBSH). Results are expressed as the mean ± standard error of the mean (SEM) ($n = 3$). Different letters represent significant differences ($p < 0.05$) between groups using one-way analysis of variance (ANOVA) and Duncan's multiple-range test. The same letters indicate $p > 0.05$.
